# Amino Acid Flux from Metabolic Network Benefits Protein Translation: the Role of Resource Availability

**DOI:** 10.1038/srep11113

**Published:** 2015-06-09

**Authors:** Xiao-Pan Hu, Yi Yang, Bin-Guang Ma

**Affiliations:** 1 Agricultural Bioinformatics Key Laboratory of Hubei Province, College of Informatics, Huazhong Agricultural University, Wuhan 430070, China

## Abstract

Protein translation is a central step in gene expression and affected by many factors such as codon usage bias, mRNA folding energy and tRNA abundance. Despite intensive previous studies, how metabolic amino acid supply correlates with protein translation efficiency remains unknown. In this work, we estimated the amino acid flux from metabolic network for each protein in *Escherichia coli* and *Saccharomyces cerevisiae* by using Flux Balance Analysis. Integrated with the mRNA expression level, protein abundance and ribosome profiling data, we provided a detailed description of the role of amino acid supply in protein translation. Our results showed that amino acid supply positively correlates with translation efficiency and ribosome density. Moreover, with the rank-based regression model, we found that metabolic amino acid supply facilitates ribosome utilization. Based on the fact that the ribosome density change of well-amino-acid-supplied genes is smaller than poorly-amino-acid-supply genes under amino acid starvation, we reached the conclusion that amino acid supply may buffer ribosome density change against amino acid starvation and benefit maintaining a relatively stable translation environment. Our work provided new insights into the connection between metabolic amino acid supply and protein translation process by revealing a new regulation strategy that is dependent on resource availability.

Protein translation is one of the most important processes to cellular life. Recent advances have extended our knowledge about the translation process in many aspects. For example, it was observed that protein expression level was mainly determined by the folding energy near the ribosome binding sites in a synthetic GFP gene library^1^ but ribosomal allocation and protein level are also affected by codon bias of the whole mRNA sequence^2^. Rare codons are preferred at the gene start^3^ probably due to various reasons including: reducing ribosomal collisions and jamming^3^,^4^, co-translational folding of the first domain of proteins^5^,^6^, chaperon requirement^7^, and the selection of purines at the first codons for the suppression of mRNA secondary structure to promote ribosome binding^8^,^9^. With the development of a new technique, ribosome footprint profiling, translation process can be captured in single nucleotide resolution^10^,^11^. Gingold and Pilpel reviewed recently that codon bias^12^, the folding energy and secondary structure of mRNA^1^,^2^,^8^,^9^, the abundance of tRNA^2^,^13^ and the codon order^14^ all have impact on translation efficiency^15^.

Most of the previous studies concentrated on the role of mRNA in the protein translation. As to amino acids, the building blocks of proteins, their role in the protein translation deserves equivalent attention. Several reports have revealed that highly expressed proteins are always rich in less costly amino acids^16^,^17^,^18^ and this property has been attributed to the requirement of higher metabolic efficiency of highly expressed genes^16^,^17^. However, the role of amino acid supply from metabolic network in protein translation remains elusive. Considering the fact that the substrate uptake rate is limited for a cell, the biosynthetic amount of amino acids used for protein translation must be restricted. Meanwhile, different proteins have different amino acid compositions but they are supported by the same metabolic network in a living organism, how would the amino acid flux (supply) from the same metabolic network affect the translation efficiency of each individual protein with different residue composition? A conceivable scenario is that a sufficient supply of amino acids from the metabolic pathways will benefit protein translation. However, this conjecture needs to be tested and quantitatively characterized.

In this post-genomic era, genome-scale metabolic networks have been constructed for many prokaryotes and eukaryotic organisms^19^. The availability of these metabolic reconstructions gives the possibility to study the role of amino acid metabolism in translation process. Meanwhile, Flux Balance Analysis (FBA) becomes popular for analyzing metabolic pathways^20^,^21^. Without the need of detailed kinetic parameters for biochemical reactions, FBA could give experimentally verifiable predictions for some phenotypes with reasonable accuracy^21^,^22^. FBA was adopted for the present analysis due to its simplicity and powerfulness. To quantitatively characterize the protein translation process, we adopted two indicators. The first one is translation efficiency (TE), defined as the ratio between the protein abundance and the corresponding mRNA level (called “local translation efficiency” by Tuller *et al.*^2^). Translation efficiency indicates the yield of protein per unit of mRNA (without considering degradation). The other one is ribosome density (RD), defined as the ratio of ribosome footprints to mRNA fragments (called “translation efficiency” by Ingolia *et al.*^10^). Ribosome density means the amount of bounded ribosome per unit of mRNA. Although both of these two indicators have been called “translation efficiency” before, they have different meanings. TE represents the final output of translation (how many proteins are produced per mRNA) while RD depicts the process of translation (how many ribosomes are involved per mRNA). In some cases, the changes of TE and RD are even in opposite direction; for example, it was observed that RD increased while protein production (TE) decreased under amino acid starvation in yeast^23^. Since TE and RD emphasize different aspects of protein translation process, both these two indicators were employed.

By using FBA, we estimated the amino acid supply for each protein in the proteomes of *Escherichia coli* and *Saccharomyces cerevisiae*. Integrated with the gene expression and ribosome profiling data, we provided a detailed description of the role of amino acid supply in translation. Our results showed that amino acid supply positively correlates with TE and RD in both *E. coli* and *S. cerevisiae*, indicating that amino acid supply facilitates translation. Furthermore, using a rank-based regression model, we revealed that amino acid supply promotes ribosome utilization. Finally, we compared the RD change between normal and amino-acid-starvation conditions and found that RD change of well-amino-acid-supplied (WAAS) genes is smaller than that of poorly-amino-acid-supplied (PAAS) genes. With these results, we proposed that amino acid supply may buffer RD change against amino acid starvation and benefit maintaining a relatively stable translation environment. Our study clarified the protein translation process in terms of the connection between metabolic amino acid supply and the efficiency of protein production.

## Results and Discussion

### Estimation of amino acid supply from metabolic flux for each individual protein

Based on the genome-scale metabolic networks for *E. coli* (model: *i*JO1366)^24^ and for *S. cerevisiae* (model: Yeast version 6)^25^, we adopted the FBA approach to estimate the amino acid supply from metabolic pathways for each individual protein. Modeled as an optimization problem, a key respect of FBA is to determine the objective function. Usually, the maximization of the production of biomass is used as the objective to predict bacterial growth rate^26^,^27^. However, such an objective function is not directly usable for our analysis. Inspired by the ME-Model^28^, we used a two-step optimization strategy to estimate amino acid supply.

**Figure 1:**
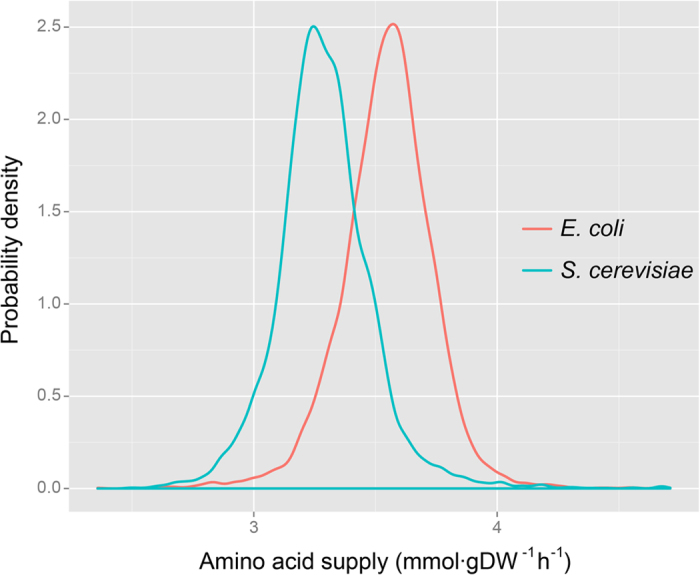
The distribution of amino acid supply for the proteins in the proteomes of *E. coli* and *S. cerevisiae*.

In the first step, the biomass reaction (*v*_biomass_) was adopted for simulating the maximum growth rate *μ* under the glucose uptake rate of 10 mmol · gDW^−1^h^−1^ (**Equation** 1). In the second step, we divided the biomass reaction into two parts: partial-biomass reaction and protein synthesis reaction. The partial-biomass reaction contains a basal level (e.g. 30%) of amino acids synthesis (the stoichiometry of amino acids was set to 30% of the original ones in biomass) and keeps the stoichiometry of all the other compounds unchanged. All the proteins shared the same partial-biomass reaction. The protein synthesis reaction is protein-specific and only contains amino acids whose stoichiometry varies according to amino acid frequency of the studied protein. In the second step, the flux value of the partial-biomass reaction (*v*_*partial-biomass*_) was set to the maximum growth rate *μ* obtained from step 1 to ensure that the organism grows under a reasonable fixed rate. Then, the flux of protein synthesis reaction (*v*_*protein*_) was optimized under the glucose uptake rate of 10 mmol · gDW^−1^h^−1^ to estimate the maximum supply of amino acid (**Equation** 2). Through the two-step optimization, we estimated amino acid supply for each protein in both *E. coli* and *S. cerevisiae* (Fig. 1). The framework of the computation procedure, with a “toy protein” as an example, was presented in the **Supplementary Figure S1**.

The two-step optimization formulism is as follows:
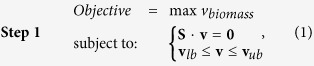
and
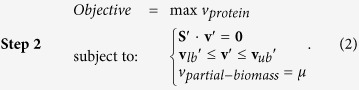


In the above formula, *v*_*biomass*_ is the flux of biomass reaction, **S** (**S’**) is the stoichiometry matrix for the involved reactions and **v**_*lb*_/**v**_*ub*_ (**v**_*lb*_’/**v**_*ub*_’) are the lower/upper bounds for the corresponding flux, respectively. The values of **v**_*lb*_/**v**_*ub*_ (**v**_*lb*_’/**v**_*ub*_’) were adopted from the original model^24^,^25^. *v*_*protein*_ is the flux of protein synthesis reaction. *v*_*partial-biomass*_ is the flux of the partial-biomass reaction and *μ* is the maximum growth rate obtained from **Step 1**. Gurobi 5.0 was used for solving this optimization problem.

In the second optimization step, the fixed flux value of *v*_*partial-biomass*_ ensures that all the precursors required for growth are produced under a reasonable rate and the value of the objective function *v*_*protein*_ was defined as the indicator of the amino acid supply under this condition (namely, Amino Acid Supply = max *v*_*protein*_, in quantity). The higher the *v*_*protein*_ value, the better the amino acid supply. We calculated the amino acid supply values with a 30% basal level of amino acid synthesis in the partial-biomass reaction and used it in the subsequent analysis (**Supplementary Dataset 1**); other basal levels of amino acid synthesis do not change the results due to the linearity of our model.

### Amino acid supply positively correlates with translation efficiency and ribosome density

Previous works showed that amino acid cost influences gene expression at both mRNA and protein levels^16^,^18^. We also found that amino acid supply positively correlates with both mRNA expression level (*R* = 0.18, *p* = 3.04e-13, n = 1597, α = 0.05 for *E. coli*; *R* = 0.11, *p* = 4.74e-12, n = 3593, α = 0.05 for *S. cerevisiae*) and protein abundance (*R* = 0.28, *p* < 2.2e-16, n = 1597, α = 0.05 for *E. coli*; *R* = 0.20, *p* < 2.2e-16, n = 3593, α = 0.05 for *S. cerevisiae*) (see **Methods** and **Supplementary Figure S2**). These positive correlations showed that highly expressed genes/proteins are better supplied by the amino acid metabolic pathways. Coupled with the previous finding that highly expressed proteins are rich in low-ATP-cost amino acids^16^, our finding indicates that the amino acids of highly expressed proteins are not only cheaper in energy cost but also richer in resource supply. Then, we moved a step forward to show how amino acid supply influences translation efficiency and ribosome density.

**Figure 2:**
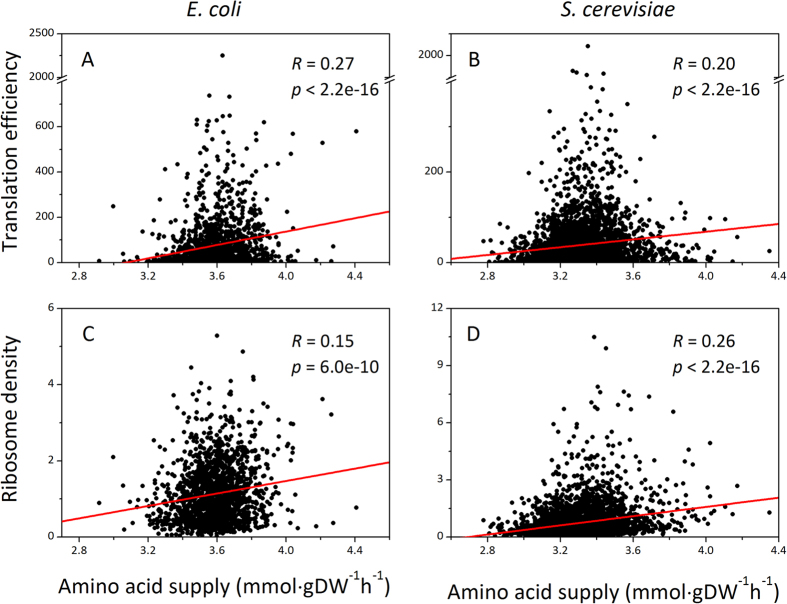
Amino acid supply positively correlates with translation efficiency and ribosome density in *E. coli* (A and C) and *S. cerevisiae* (B and D).

Firstly, we found a positive correlation between amino acid supply and translation efficiency in both *E. coli* (*R* = 0.27, *p* < 2.2e-16, n = 1597, α = 0.05) and *S. cerevisiae* (*R* = 0.20, *p* < 2.2e-16, n = 3593, α = 0.05) (Fig. 2). Then, positive correlations were also found between amino acid supply and ribosome density in both *E. coli* (*R* = 0.15, *p* = 6.04e-10, n = 1597, α = 0.05) and *S. cerevisiae* (*R* = 0.26, *p* < 2.2e-16, n = 3593, α = 0.05) (Fig. 2). These positive correlations mean that genes with better amino acid supply (higher amino acid supply value) have higher TE and RD. As both TE and RD are indicators for translation process, the higher TE and RD of genes with better amino acid supply indicate that amino acid supply facilitates translation.

### Amino acid supply contributes to the utilization efficiency of ribosomes

Since other factors such as codon usage bias and mRNA folding energy at gene start also influence translation efficiency^2^. Whether the correlation between amino acid supply and RD (or TE) could be explained by these factors should be tested. We used rank-based linear regression models^29^ to show that amino acid supply is an indispensable factor in the interpretation of both RD and TE. Firstly, we performed linear regression between RD (as the dependent variable) and independent variables that include amino acid supply, codon usage bias (indicated by Codon Adaptation Index, CAI), and mRNA folding energy at gene start (−4 to +38 from the translation start site^1^). As shown in **Supplementary Table S1**, for *E. coli*, the total correlation coefficient is *R* = 0.49 and the p-value for amino acid supply is *p* = 1.26e-3, while for *S. cerevisiae*, the total correlation coefficient is *R* = 0.57 and the p-value for amino acid supply is *p* < 2.2e-16. Both the two p-values for amino acid supply indicate that amino acid supply is an indispensable factor in explaining RD. Since RD depicts the process of translation (how many ribosomes are involved per mRNA) and TE represents the final output of translation (how many proteins are produced per mRNA), there is correlation between TE and RD (**Supplementary Figure S3**).Therefore, to show the contribution of amino acid supply to TE, we have to remove the effect of RD. By treating RD as an independent variable, we performed linear regression between TE (as the dependent variable) and the independent variables (including amino acid supply, RD, codon usage bias, and mRNA folding energy at gene start); the results showed that the total correlation coefficient is *R* = 0.64 and the p-value for amino acid supply is *p* = 1.46e-7 in *E. coli*, and the total correlation coefficient is *R* = 0.63 and the p-value for amino acid supply is *p* = 4.29e-3 in *S. cerevisiae*. From the p-values, it can be found that amino acid supply still plays an indispensable role in explaining TE. This result means that the genes with higher amino acid supply can translate more proteins than genes with lower amino acid supply under the control of all the other factors like CUB, mRNA folding energy, and RD, and validated the contribution of amino acid supply to the efficiency of ribosome utilization.

### Amino acid supply buffers RD change against amino acid starvation

**Figure 3:**
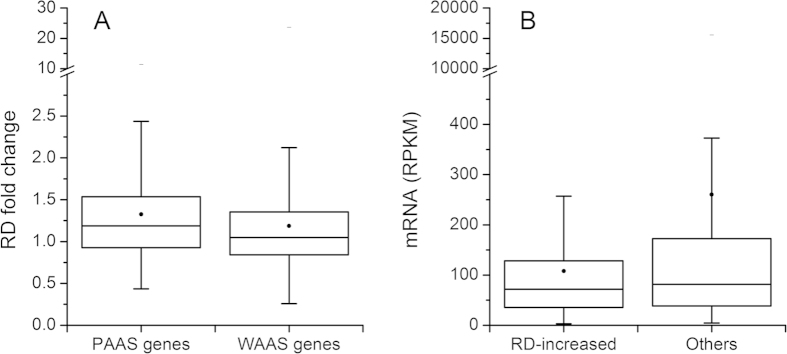
The role of amino acid supply in buffering ribosome density against amino acid starvation. (**A**) RD change of WAAS genes was significantly smaller than that of PAAS genes under amino acid starvation. As (external) amino acids starvation makes more ribosome stalling on mRNA and translation speed slowing down, better (internal) amino acid supply makes WAAS genes less affected. (**B**) RD-increased genes are lowly expressed in mRNA level. In the boxplot, the box shows the first quartile (25%), the median, and the third quartile (75%); the whisker shows the 5% or 95% percentile; the dot inside the box represents the mean value and the short horizontal bar above the whisker represents the maximum value of each data set.

For a living cell, three sources of amino acids are available for protein synthesis: transported from the external cellular environment, the biosynthetic pathways of amino acids, and the degradation pathways of proteins. What would happen to protein translation if the external source of amino acids was blocked? It has been observed recently that the RD of most genes increased under amino acid starvation in *S. cerevisiae*^10^. This phenomenon has been simulated by a theoretical model and explained as the insufficiency of (external) amino acid nutrients leads to stalling of ribosomes on the mRNA sequences^23^. How would the (internal) amino acid supply from the metabolic pathways connect to this phenomenon? We used the above RD change (fold change, RPKM_starvation_/RPKM_normal_) data under amino acid starvation in *S. cerevisiae*^10^ to explore this question. By dividing genes into two groups (WAAS and PAAS, the former with top 50% amino acid supply), the RD change was compared and it was found that the RD change of WAAS genes was significantly smaller than that of PAAS genes (*p* = 7.58e-16, one-sided Wilcoxon rank sum test, median: 1.05 vs. 1.19, n: 1401 vs. 1400, α = 0.05) under amino acid starvation (Fig. 3A and **Supplementary Dataset 2**).

The smaller RD change of WAAS genes under amino acid starvation means that less ribosome stalling on the mRNA sequence, because WAAS genes could get more and relatively sufficient amino acids (high amino acid supply) from the metabolic network compared with PAAS genes under amino acid starvation. This connection implies that a better amino acid supply could buffer the reduction of amino acid nutrients from environment and make WAAS genes less affected. Meanwhile, compared with the ribosome stalling explanation in a simulation result^23^ where all the proteins are equally affected, our result showed that this buffering effect varies in different proteins according to their corresponding amino acid supply, which is in line with the previous finding that the change of translation rate varies according to gene function under amino acid starvation^10^,^30^. Moreover, the compensation between (internal) amino acid supply and (external) amino acid nutrients also showed that amino acid supply could not only promote ribosome utilization but also buffer the RD change against the disturbance from environment.

### Amino acid supply benefits maintaining stable translation environment under amino acid starvation

As stated above, amino acid supply could buffer RD change against amino acid starvation. How would this buffering effect benefit translation? It has been shown that amino acid supply positively correlates with mRNA level (**Supplementary Figure S2**) and that the PAAS genes have larger RD increase than WAAS genes under amino acid starvation (Fig. 3A). This implies that the RD-increased genes under amino acid starvation should have low mRNA expression levels. By sifting the data, we found that the mRNA expression level of RD-increased genes (variation greater than 2.0 fold) were indeed lower than other genes (*p* = 2.78e-3, one-sided Wilcoxon rank sum test, median: 71.63 vs. 81.44, n: 235 vs. 2566, α = 0.05) (Fig. 3B and **Supplementary Dataset 2**). The lower transcription level of the RD-increased genes (mainly PAAS genes) could benefit protein translation by maintaining a relatively stable free ribosome pool.

**Figure 4:**
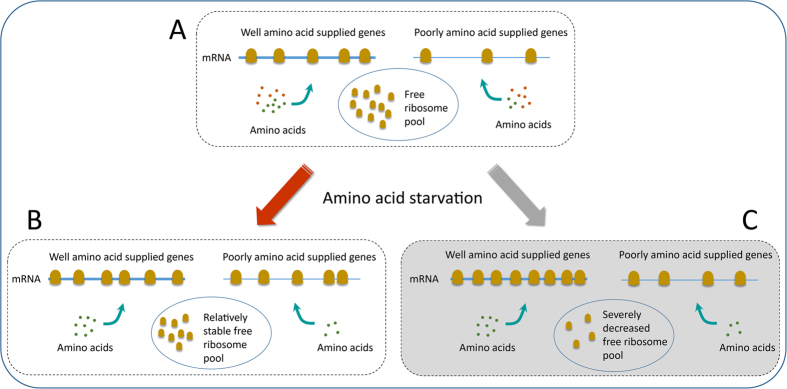
The role of amino acid supply in maintaining a relatively stable translation environment under amino acid starvation. (**A**) Under normal condition, well-amino-acid-supplied (WAAS) genes (indicated by more green dots representing biosynthetic amino acids) have higher mRNA expression level (indicated by the thicker line representing mRNA) and higher ribosome density (indicated by more ribosomes on the mRNA line) than poorly amino acid supplied (PAAS) genes (indicated by less green dots representing biosynthetic amino acids) and the free ribosome pool has a fix size. (**B**) Under amino acid starvation, the amino acids transported from environment (indicated by red dots) decrease acutely (removed in the illustration), the WAAS genes have smaller increase of ribosome density (indicated by the smaller increase of ribosome number on the mRNA line) than the PAAS genes (indicated by a larger increase of ribosome number on the mRNA line). As PAAS genes are of low expression levels, the significant increase of ribosome density of PAAS genes has a relatively smaller influence on the free ribosome pool size (indicated by the small change of the ribosome number). (**C**) If ribosome-density-increased genes were WAAS genes that are highly expressed in mRNA level, more ribosome would stall on mRNA and the size of the free ribosome pool would decrease sharply. Since free ribosomes play an important role in translation initiation, amino acid supply buffers the change of ribosome density and keeps the number of free ribosomes so as to maintain the cell in a stable translation environment under amino acid starvation.

The mechanism was illustrated in Fig. 4. As shown, under normal condition (Fig. 4A), WAAS genes have higher transcription expression level and higher RD than PAAS genes and the free ribosome pool has a fixed size. When the stress of amino acid starvation is applied (Fig. 4B), the transported amino acids from the external environment decrease acutely and the amino acids synthesized by metabolic network increase by up-regulation of the genes for amino acid biosynthesis. The WAAS genes are less affected by the starvation due to a better (internal) amino acid supply than the PAAS genes (buffering effect), which helps to the maintenance of their ribosome density at a relatively stable level (smaller RD increase). Therefore, the genes with significant RD increase are mainly the PAAS genes that are lowly expressed in mRNA levels. The low expression of the RD-increased genes (mainly PAAS genes) leads to less ribosome stalling on the mRNA sequence, resulting in a relatively stable size of the free ribosome pool. Suppose the RD-increased genes were highly expressed in transcription level (Fig. 4C), more ribosomes would stall on the mRNA sequences and the number of free ribosomes would decrease severely, which is very harmful for cell surviving. Since the size of free ribosome pool plays an important role in translation initiation^23^, the fact that RD-increased genes have low transcription levels could ensure that the translation can proceed under a relatively stable environment. In addition, the production of ribosomes accounts for a substantial proportion of total biomass. The occurrence of more stalling ribosomes means a severe resource waste. Therefore, the buffering effect of amino acid supply for RD change would not only optimize ribosome allocation but also benefit maintaining a relatively stable translation environment for the cell under amino acid starvation. This mechanism also demonstrates the different regulation strategies in transcription (mRNA) and translation (ribosome density) in response to amino acid starvation in that the mRNA expression level is mainly regulated by transcription factors while ribosome density is directly regulated by the degree of resource (amino acid) availability.

## Conclusion

In this work, based on the genome-scale metabolic network, we estimated the amino acid supply (flux) for each protein in the proteomes of *E. coli* and *S. cerevisiae*. We found that amino acid supply facilitates translation efficiency and ribosome utilization. The results also showed that the change of ribosome density is regulated, to some extent, by amino acid supply in response to amino acid starvation. The better amino acid supply for the highly expressed genes ensures a relatively stable translation environment for the cell and contributes to the survival of an organism.

## Methods

### Genome sequence and metabolic network

The genome and proteome sequences of *E. coli* K-12 MG1655 and *S. cerevisiae* S288C were downloaded from NCBI^31^ and SGD database^32^, respectively. The used genome-scale metabolic network constructions were *i*JO1366 for *E. coli*^24^ and Yeast-6 for *S. cerevisiae*^25^.

### CAI calculation

Using ribosome proteins as the default highly expressed genes, the “cusp” and “cai” programs in the EMBOSS package^33^ were employed for the calculation of codon usage table and codon adaptation indices (CAI)^12^ for both *E. coli* and *S. cerevisiae*.

### Folding energy calculation

ViennaRNA package^34^ were used for calculating mRNA folding energy at gene start (−4 to +38 from translation start site, 42 bp in length).

### Correlation and regression

The correlations in this work were the nonparametric Spearman correlation calculated by R scripts.

Rfit package^29^ in R was used to perform the rank-based linear regression analysis.

### Translation efficiency


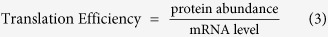


Translation efficiency was defined as the ratio between the protein abundance and the corresponding mRNA expression level. In order to make the data more reliable, both protein abundance and mRNA expression level used in this study were based on several works. Protein abundance for *E. coli* and *S. cerevisiae* were obtained from PaxDb (version 3.0)^35^ and mRNA expression data were adopted from Lu’s work^36^. The mRNA expression levels from Lu’s work were the average expression level from several previous works. Only the genes with mRNA expression level greater than 0.5 molecules/cell were selected to ensure reliability.

### Ribosome density


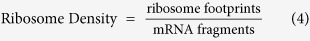


Ribosome density was defined as the ratio of the number of ribosome footprints to the number of mRNA fragments. Although ribosome occupancy varies along a gene sequence^10^,^11^, the variation has small effects on the average RD^37^. Therefore, the RD for each gene was used.

For *S. cerevisiae*, the ribosome footprints and mRNA fragments were obtained from Ingolia^10^. The expression level data (RPKM) for both species were obtained from Gene Expression Omnibus (GEO) under accession number GSE13750. For *E. coli*, the ribosome footprint and mRNA fragment data were obtained from Li^37^ under GEO accession number GSE53767.

Ribosome footprints and mRNA fragments (RPKM) estimation procedure is as follows:

Fastx_clipper from FASTX-Toolkit (http://hannonlab.cshl.edu/fastx_toolkit/) was used to clip linkers CTGTAGGCACCATCAAT and only reads with size between 20 and 42 nucleotides were retained^37^.

Bowtie 1.0^38^ was used for aligning trimmed reads to non-coding RNA reference (NC_000913.frn, version 2) to discard non-coding RNA.

Bowtie 1.0 was used for aligning the rest RNA reads to the genomic reference (NC_000913.fna, version 2).

Samtools^39^ was used for the sorting of aligned reads and cufflinks^40^ was used to calculate the expression level (RPKM) for each gene.

For both the ribosome density and mRNA fragments expression level (RPKM), only genes with the average RPKM value greater than 1 and variation <1.5 fold between replicates were retained. Both the ribosome density and mRNA fragments expression level (RPKM) were the average value among the replicates.

## Additional Information

**How to cite this article**: Hu, X.-P. *et al.* Amino Acid Flux from Metabolic Network Benefits Protein Translation: the Role of Resource Availability. *Sci. Rep.* **5**, 11113; doi: 10.1038/srep11113 (2015).

## Supplementary Material

Supplementary Information

Supplementary Information

Supplementary Information
