# Supplementary material for: Amino Acid Flux from Metabolic Network Benefits Protein Translation: the Role of Resource Availability
Source: Sci Rep. 2015 Jun 9;5:11113. doi: 10.1038/srep11113 (PMC4460870; doi:10.1038/srep11113)
Supplement: Supplementary Information [file srep11113-s1.pdf]

Supplementary Information:

Amino Acid Flux from Metabolic Network Benefits Protein Translation: the Role of Resource Availability

Xiao-Pan Hu, Yi Yang, Bin-Guang Ma*

Agricultural Bioinformatics Key Laboratory of Hubei Province, College of Informatics, Huazhong Agricultural University, Wuhan 430070, China

* E-mail: mbg@mail.hzau.edu.cn

Figure S1. The framework of amino acid supply estimation procedure.

Figure S2. Positive correlation between amino acid supply and gene expression.

Figure S3. The relationship between ribosome density and translation efficiency.

Table S1. Rand-based linear regression models for RD and TE.

Supplementary Dataset 1. Amino acid supply, translation efficiency and ribosome density data used in this work.

Supplementary Dataset 2. The ribosome density change (RD-change) under amino acid starvation for the analyzed *S. cerevisiae* genes.

Figure S1. The framework of amino acid supply estimation procedure.

Step 1. Estimate growth rate μ

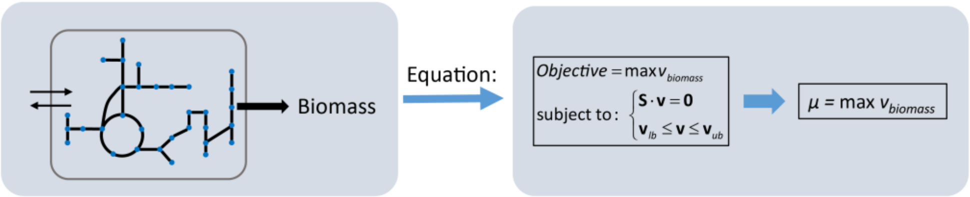

The growth rate μ of the organism was estimated by maximizing the flux of biomass reaction ($v_{biomass}$).

Step 2. Estimate amino acid supply (AAS)

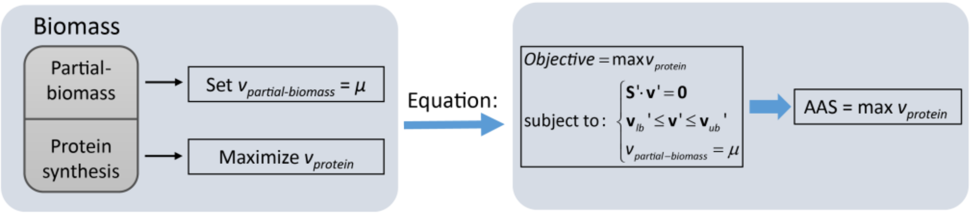

The biomass reaction was divided into partial-biomass and protein synthesis reaction.

The partial-biomass reaction contains a basal level (e.g. 30%) of amino acids synthesis (the stoichiometry of amino acids was set to 30% of the original ones in biomass) and keeps the stoichiometry of all the other compounds unchanged (the same to the original ones in the biomass function). Then, the flux of partial-biomass reaction ($v_{partial\text{-}biomass}$) was set to μ to ensure the organism grows under a reasonable and fixed rate. All the proteins shared the same partial-biomass reaction.

The protein synthesis reaction is protein-specific and only contains amino acids whose stoichiometry varies according to amino acid frequency of the studied protein. Here, we present a toy protein A as an example to show how to estimate amino acid supply. The amino acid frequency of protein A was shown in the table below.

Amino acid	Frequency
Ala-L	9%
Arg-L	3%
Asn-L	5%
...	...
Val-L	11%

Based on the amino acid frequency, we can get the protein synthesis reaction of protein A:

0.09 Ala-L + 0.03 Arg-L + 0.05 Asn-L + ... + 0.11 Val-L => protein A

By maximizing the flux of protein synthesis reaction (here, $v_{protein\text{-}A}$), we can get the amino acid supply of protein A (AAS_A, $AAS_A = \max v_{protein\text{-}A}$).

Through the two-step optimization, we get the amino acid supply for protein A (AAS_A). By applying the second step for each protein in the proteome, we get the amino acid supply for all the proteins.

Figure S2. Positive correlation between amino acid supply and gene expression. Amino acid supply significantly correlates with mRNA expression level and protein abundance in both *E. coli* (A and C) and *S. cerevisiae* (B and D).

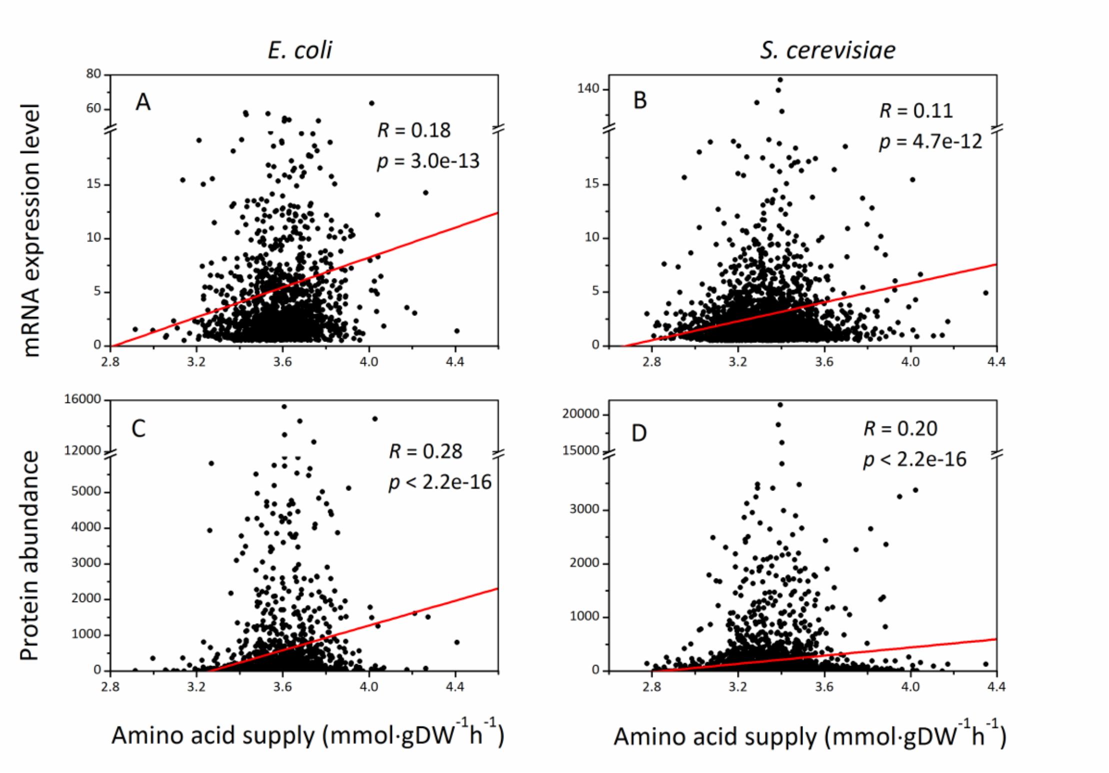

Figure S3. The relationship between ribosome density and translation efficiency.

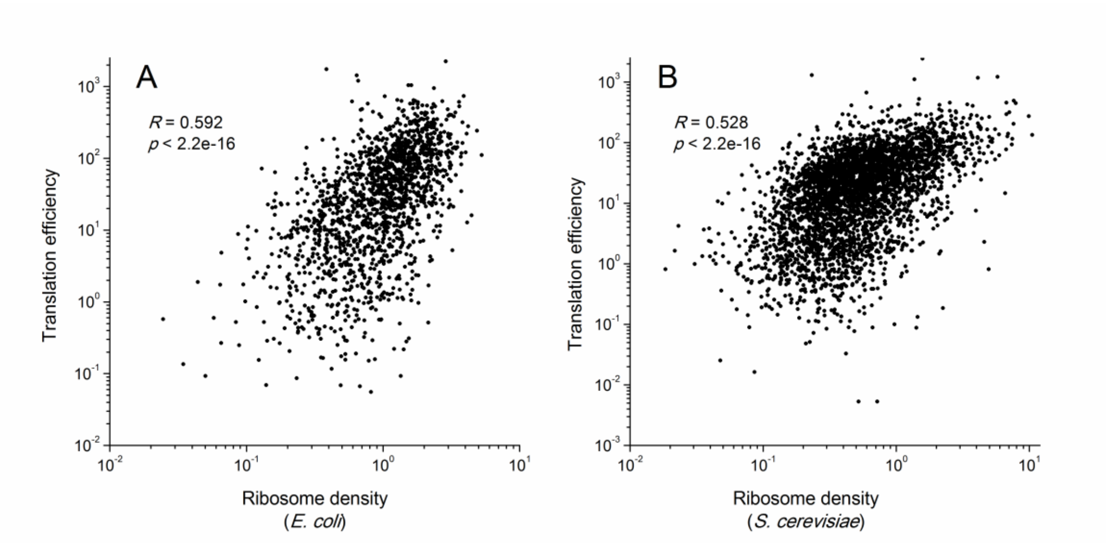

Table S1. Rank-based linear regression models for RD and TE

(A) The rank-based linear regression model for RD in *E. coli* [a]

	Estimate	p-value
Intercept	-1.27	3.03e-4
CAI	2.90	2.2e-16
AAS	0.32	1.26e-3
FE	0.04	1.46e-14

[a] Multiple $R = 0.49$ and $p < 2.2e\text{-}16$.

(B) The rank-based linear regression model for TE in *E. coli* [b]

	Estimate	p-value
Intercept	-226.40	2.2e-16
RD	27.42	2.2e-16
CAI	250.84	2.2e-16
AAS	35.90	1.46e-7

[b] Multiple $R = 0.64$ and $p < 2.2e\text{-}16$.

(C) The rank-based linear regression model for RD in *S. cerevisiae* [c]

	Estimate	p-value
Intercept	-2.03	2.2e-16
CAI	2.67	2.2e-16
AAS	0.57	2.2e-16

[c] Multiple $R = 0.57$ and $p < 2.2e\text{-}16$.

(D) The rank-based linear regression model for TE in *S. cerevisiae* [d]

	Estimate	p-value
Intercept	-38.42	1.35e-8
RD	17.57	2.2e-16
CAI	121.03	2.2e-16
AAS	5.91	4.29e-3

[d] Multiple $R = 0.63$ and $p < 2.2e\text{-}16$.
